# Migration-related factors and mental health among international students in a UK higher education setting: a cross-sectional study

**DOI:** 10.1186/s12889-026-28749-4

**Published:** 2026-08-01

**Authors:** Mandu Stephen Ekpenyong, Umme Kulsuma Diba

**Affiliations:** 1 School of Nursing and Public Health, Manchester Metropolitan University, Manchester, UK; 2 School of Health and Society, University of Wolverhampton, Wolverhampton, UK

**Keywords:** International students, Mental health, Depression, Acculturation, Perceived stress

## Abstract

**Background:**

International students may experience psychological challenges associated with migration, academic demands, and adjustment to a new cultural environment. Although previous research has documented mental health concerns in this population, less is known about the relationships among migration-related factors, perceived stress, and mental health outcomes within specific UK higher-education settings.

**Methods:**

A cross-sectional quantitative study was conducted among international students enrolled at the University of Wolverhampton. Data were collected using an online questionnaire that included demographic information, migration-related variables, the Depression Anxiety Stress Scale (DASS-21), and a measure of perceived mental stress. Descriptive statistics were used to summarise participants’ characteristics and mental health outcomes. Inferential analyses included independent samples t tests, one-way ANOVA, Pearson’s correlation, and multiple linear regression to assess predictors of depression scores, including perceived mental stress, length of stay in the UK, gender, and region of origin.

**Results:**

A total of 115 international students participated. The sample included 73 women (63.5%), 41 men (35.7%), and one participant who preferred not to report gender (0.9%). Mean scores were 1.14 (*SD* = 0.58) for depression, 0.98 (*SD* = 0.55) for anxiety, and 1.19 (*SD* = 0.56) for stress. Men reported higher mean depression, anxiety, and stress scores than women; however, these results should be interpreted cautiously because of the exploratory design and unequal gender groups. No statistically significant differences were identified across regions of origin, and length of stay in the UK was not significantly related to the mental health outcomes. In the regression model, perceived mental stress and gender were associated with depression scores, whereas length of stay and region of origin were not.

**Conclusion:**

In this single-institution, cross-sectional sample, perceived mental stress and gender were associated with depression scores, whereas length of stay and region of origin were not. These findings are preliminary and do not establish causality. They support the need for larger, multi-institutional, and longitudinal studies to test whether the observed relationships are reproducible and to clarify the mechanisms involved before recommending specific policy or practice changes.

**Supplementary Information:**

The online version contains supplementary material available at https://doi.org/10.1186/s12889-026-28749-4.

## Background

International students constitute a substantial and growing proportion of the higher education population in the United Kingdom, contributing to academic diversity, institutional income, and the broader economy [1]. Evidence indicates that some international students experience substantial psychological distress during university study [2–4]. Depression, anxiety, and stress have been repeatedly documented in student and international-student populations and are relevant because they may coincide with poorer academic functioning, reduced well-being, and difficulties in longer-term adjustment [2–4]. However, prevalence estimates vary across studies, measures, institutions, and cultural groups; therefore, international students should not be treated as a homogeneous high-risk population.

This study is grounded in Berry’s acculturation framework [5, 6]. The framework conceptualises adaptation as a dynamic process arising from contact between cultural contexts and distinguishes the experience of acculturative stress from the mere fact or duration of migration. Applied to international students, it predicts that psychological outcomes are more plausibly related to the appraisal of academic, financial, social, linguistic, and cultural demands, and to the resources available to manage them, than to the region of origin or length of residence alone [5–8]. This framing also cautions against interpreting demographic characteristics as direct causes of distress: gender, region, and length of stay may mark different exposures or resources.

Within the UK context, concern about international students’ well-being has increased in relation to living costs, visa uncertainty, social isolation, and access to culturally appropriate support [3, 9, 10]. The present study, therefore, examines whether perceived migration-related stress, gender, region of origin, and length of stay are associated with depression, anxiety, and stress within one post-1992 UK university. The acculturation framework provides the analytical logic: perceived stress is treated as a proximal appraisal of current demands, while length of stay and region are treated as comparatively distal contextual indicators. Because the design is exploratory and cross-sectional, the analysis estimates relationships rather than effects and is intended primarily to generate hypotheses for subsequent research.

## Objectives of the study

The objectives were to: (1) describe depression, anxiety, stress, and perceived mental stress; (2) assess whether mental health scores differed by gender or region of origin; (3) examine associations between length of stay, perceived mental stress, and mental health outcomes; and (4) determine whether perceived mental stress, length of stay, gender, and region of origin were independently associated with depression scores.

### Research questions

- RQ1: What levels of depression, anxiety, stress, and perceived mental stress were reported in this sample?
- RQ2: Were depression, anxiety, and stress scores different across gender and regional groups?
- RQ3: How were the length of stay and perceived mental stress related to depression, anxiety, and stress?
- RQ4: Which of the measured variables were independently associated with depression scores in the multivariable model?

## Methods

### Study design and setting

A quantitative cross-sectional study design was employed to examine the relationship between migration-related stressors and mental health outcomes among international students. This cross-sectional design allowed examination of relationships and associations among variables but did not allow causal inferences. The study was conducted at the University of Wolverhampton, a post-1992 university in the United Kingdom with a diverse international student population.

### Participants and sampling

Participants were international students aged 18 years or older who were enrolled in undergraduate or postgraduate programmes at the University of Wolverhampton. Convenience sampling was used to recruit participants through online platforms, including university mailing lists and social media groups. Students who self-identified as domestic or home students were excluded. The use of convenience sampling may have introduced selection bias and limited the generalisability of the findings beyond the study sample.

A sample of 100–150 participants was judged pragmatically adequate for this exploratory, single-institution study; however, no a priori power calculation was reported. A total of 115 participants completed the questionnaire and were included in the analysis. The absence of a formal power calculation, together with small or uneven subgroup sizes, limits confidence in the null findings and should be considered when interpreting the ANOVA and regression estimates.

## Measures

### Mental health outcomes

Mental health was assessed using the 21-item Depression Anxiety Stress Scales (DASS-21), a widely used self-report instrument comprising three seven-item subscales that measure depression, anxiety, and stress. Each item is rated on a four-point Likert scale ranging from 0 to 3, with higher scores indicating greater symptom severity. Internal consistency was evaluated separately for each subscale because a single reliability coefficient for the overall instrument does not adequately reflect the reliability of its distinct constructs. In the present study, the DASS-21 demonstrated good internal consistency, with Cronbach’s alpha coefficients of 0.823 for the Depression subscale, 0.754 for the Anxiety subscale, and 0.799 for the Stress subscale. The overall 21-item scale also demonstrated excellent internal consistency (Cronbach’s *α* = 0.92).

### Migration-related stressors

Migration-related mental stress was assessed using a study-specific self-report questionnaire developed from the literature on acculturative stress among international students. The instrument included items covering key domains of migration-related stress, including financial difficulties, academic pressure, social isolation, language barriers, and cultural adjustment. Responses were recorded on a Likert scale and averaged to generate a composite measure of perceived migration-related mental stress, with higher scores indicating greater perceived stress. The migration-related mental stress composite demonstrated good internal consistency in the present sample (Cronbach’s α = 0.804). As this was a study-specific instrument, internal consistency provides evidence of reliability but does not establish construct validity. Consequently, the findings relating to this measure should be interpreted as exploratory, and future research should further evaluate its dimensionality, convergent validity, and measurement performance across culturally diverse student populations. An English version of the questionnaire is provided as Supplementary File 1.

### Data collection procedure

Data were collected using an anonymous online survey hosted on Microsoft Forms. Participants were provided with an information sheet outlining the purpose of the study, the confidentiality measures, and their right to withdraw at any time, in accordance with ethical guidelines. Informed consent was obtained electronically before participation.

### Statistical analysis

Data were analysed using statistical software. An anonymised dataset and selected SPSS outputs are provided in the appendices. Descriptive statistics summarised participant characteristics and study variables. Assumptions of normality were assessed using the Shapiro–Wilk test and visual inspection of histograms and Q–Q plots; homogeneity of variance was assessed with Levene’s test. Independent-samples t tests and one-way ANOVA examined mean differences by gender and region, respectively. Pearson’s correlations assessed bivariate relationships among length of stay, perceived mental stress, depression, anxiety, and stress. Multiple linear regression was used to examine whether perceived mental stress, length of stay, gender, and region of origin were independently associated with depression scores. Depression was selected a priori as the dependent variable because of its theoretical relevance to chronic and acculturative stress; this choice should not be interpreted as evidence that depression was caused by the included predictors.

Statistical significance was set at *p* < .05. Regression assumptions, including linearity, independence of errors, normality of residuals, homoscedasticity, and absence of multicollinearity, were examined and met.

## Results

### Demographic characteristics of study participants

The demographic characteristics of the study participants are summarised in Table 1. A total of 115 international students participated. The sample included 73 women (63.5%), 41 men (35.7%), and one participant who preferred not to report their gender (0.9%). The largest age groups were 28–32 years, comprising 46 participants (40.0%), and 23–27 years, comprising 45 participants (39.0%). Regional representation included 41 participants from African countries (35.6%), 38 from Bangladesh (33.1%), and 36 from other Asian countries (31.4%). Most participants were enrolled in Public Health programmes, 87 (76.0%), followed by International Relations, 23 (20.0%), and other programmes, 5 (4.0%). Most participants had lived in the UK for 6–12 months, 83 (72.2%); 16 (13.9%) had lived in the UK for less than 6 months, and 16 (13.9%) for more than 12 months. All 115 participants (100.0%) reported education as their reason for migration. The narrow distribution of programmes, reasons for migration, and length of stay, together with the uneven subgroup sizes, restricts between-group comparisons and reduces the generalisability of the findings.

**Table 1:** Demographic characteristics of participants (*n* = 115)

Variable	*n*	%
Gender		
Man	41	35.7%
Woman	73	63.5%
Prefer not to say	1	0.9%
Age group (years)		
18–22	3	3.0%
23–27	45	39.0%
28–32	46	40.0%
≥ 32	21	18.0%
Nationality / Region		
Bangladesh	38	33.1%
Other Asian countries	36	31.4%
African countries	41	35.6%
Middle East	0	0
Other	0	0
Programme of study		
Public Health	87	76.0%
International Relations	23	20.0%
Other	5	4.0%
Length of stay in the UK		
< 6 months	16	13.9%
6–12 months	83	72.2%
> 12 months	16	13.9%
Reason for migration		
Education	115	100.0%
Work	0	0
Family	0	0
Other	0	0

### Descriptive statistics of study variables

Descriptive statistics for depression, anxiety, stress, and mental stress are presented in Table 2. Participants reported moderate levels of depression (*M* = 1.14, *SD* = 0.58), anxiety (*M* = 0.98, *SD* = 0.55), and stress (*M* = 1.19, SD = 0.56). The mean mental stress score was 2.91 (*SD* = 0.51), indicating moderate perceived stress related to migration experiences.

**Table 2:** Descriptive statistics

	*N*	minimum	maximum	mean	Std. deviation
Depression	115	0.00	3.00	1.1352	0.57577
Anxiety	115	0.00	2.00	0.9843	0.54864
Stress	115	0.00	2.71	1.1919	0.56400
Mental Stress	115	1.62	4.85	2.9148	0.51067
Valid *N*(listwise)	115				

Mean scores are reported on their original scale ranges, with DASS-21 items scored from 0 to 3 and mental stress items scored from 1 to 5, where higher scores indicate greater symptom severity or stress.

### Gender differences in mental health outcomes

Independent samples *t*-*tests* were conducted to examine gender differences in depression, anxiety, and stress scores. As shown in Table 3, statistically significant gender differences were observed across all three mental health outcomes, with male participants reporting higher scores than female participants. Male participants reported significantly higher levels of depression (*M* = 1.33) compared with female participants (*M* = 1.02), *t*(112) = 2.90, *p* = .005. Similarly, anxiety scores were significantly higher among men (*M* = 1.12) than women (*M* = 0.90), *t*(112) = 2.11, *p* = .037. For stress, male participants again reported higher mean scores (*M* = 1.38) compared with female participants (*M* = 1.08), with the difference reaching statistical significance, *t*(112) = 2.76, *p* = .007. Overall, the observed gender differences ranged from small-to-moderate to moderate in magnitude, indicating that the statistically significant findings also reflect meaningful differences in mental health outcomes between male and female international students.

**Table 3:** Independent samples test comparing depression, anxiety, and stress scores by gender

Outcome	Mean (Men)	Mean (Women)	t(df)	*p*
Depression	1.33	1.02	2.90(112)	0.005
Anxiety	1.12	0.90	2.11(112)	0.037
Stress	1.38	1.08	2.76(112)	0.007

### Regional differences in mental health outcomes

One-way analyses of variance (ANOVA) were conducted to inspect differences in depression, anxiety, and stress scores across different regional groups. As shown in Table 4, no statistically significant differences were observed for depression, *F*(4,107) = 1.67, *p* = .162, anxiety, *F*(4,107) = 1.42, *p* = .231, or stress, *F*(4,107) = 1.28, *p* = .284. The absence of statistically significant regional differences could have reflected uneven group sizes, which reduced statistical power and limited the detection of actual group effects.

**Table 4:** One-way ANOVA comparing mental health outcomes across regional groups

Outcome	df (between, within)	F	*p*
Depression	4,107	1.67	0.162
Anxiety	4,107	1.42	0.231
Stress	4,107	1.28	0.284

### Associations between length of stay and mental health outcomes

Pearson’s correlation analysis was conducted to examine the associations between length of stay in the UK and mental health outcomes. As shown in Table 5, depression, anxiety, stress, and mental stress were all significantly and positively correlated with one another, indicating substantial overlap among these mental health constructs.

**Table 5:** Pearson correlations between mental health variables and length of stay

Variable	1	2	3	4	5
Depression	-				
Anxiety	0.75**	-			
Stress	0.82**	0.79**	-		
Mental Stress	0.61**	0.41**	0.57**	-	
Length of stay	0.08	0.02	0.07	0.06	-

**p < .01 (two-tailed)

Depression showed a strong positive correlation with anxiety (*r* = .75, *p* < .01) and stress (*r* = .82, *p* < .01), as well as a moderate positive correlation with mental stress (*r* = .61, *p* < .01). Anxiety was also strongly correlated with stress (*r* = .79, *p* < .01) and showed a moderate association with mental stress (*r* = .41, *p* < .01). In addition, stress was moderately and positively correlated with mental stress (*r* = .57, *p* < .01).

In contrast, length of stay in the UK was not significantly associated with any of the mental health outcomes, including depression (*r* = .08), anxiety (*r* = .02), stress (*r* = .07), or mental stress (*r* = .06), with all correlations being weak and non-significant. These findings suggest that the duration of residence in the UK was not related to levels of psychological distress in this sample. Length of stay in the UK was coded numerically to reflect increasing duration of residence and analysed as a continuous variable in the correlation analyses.

### Predictors of depression

Multiple linear regression analysis was conducted to identify predictors of depression scores among international students. The overall regression model was statistically significant, *F*(4, 106) = 17.39, *p* < .001, and explained approximately 40% of the variance in depression scores. As shown in Table 6, mental stress emerged as the strongest statistical association with depression, with higher levels of mental stress being associated with higher depression scores (*B* = 0.624, *SE* = 0.085, *β* = 0.561, *p* < .001). This indicates that, holding other variables constant, a one-unit increase in mental stress was associated with a 0.624 increase in depression scores. Gender was also a statistically significant predictor of depression (*B* = − 0.203, *SE* = 0.090, *β* = −0.174, *p* = .026), suggesting that depression scores differed significantly by gender. The negative regression coefficient indicates that the gender category coded with the higher numerical value reported lower depression scores.

**Table 6:** Multiple linear regression predicting depression scores

Predictor	B	SE	β	*p*
Mental Stress	0.624	0.085	0.561	<0.001
Length of stay	0.037	0.080	0.035	0.643
Gender	-0.203	0.090	-0.174	0.026
Region	-0.050	0.037	-0.101	0.186

Dependent variable: depression

In contrast, length of stay in the UK (*B* = 0.037, *SE* = 0.080, *β* = 0.035, *p* = .643) and region of origin (*B* = − 0.050, *SE* = 0.037, *β* = −0.101, *p* = .186) were not significant predictors of depression in the model. These findings suggest that psychological factors, particularly mental stress, play a more substantial role in predicting depression than migration-related demographic variables in this sample of international students.

## Discussion

This exploratory cross-sectional study examined associations between migration-related factors and mental health outcomes among international students at one UK university. Participants reported mean depression, anxiety, and stress scores in the moderate range as described by the authors’ scoring approach. Men had higher mean scores than women on all three DASS-21 subscales. Region of origin and length of stay were not statistically related to the outcomes, whereas perceived mental stress and gender were independently associated with depression in the regression model. These findings address the research questions but do not show that migration-related stress or gender caused depression, nor do the non-significant results demonstrate equivalence between regional or length-of-stay groups.

### Gender differences in mental health outcomes

Men in this sample reported higher depression, anxiety, and stress scores than women. This result is analytically noteworthy because it differs from a common pattern in the wider student mental-health literature, but it should not be generalised beyond the sample. The gender groups were unequal; one participant was excluded from the binary comparison, and the study did not measure potential mechanisms such as help-seeking norms, financial responsibility, social support, discrimination, or coping. Accordingly, gender should be interpreted as an associated characteristic rather than an explanatory cause.

One plausible explanation relates to gendered norms surrounding emotional expression and help-seeking behaviours. Male students, particularly those from collectivist or patriarchal cultural contexts, may face strong social expectations to demonstrate emotional resilience and self-reliance, which can discourage help-seeking and emotional disclosure [11]. Evidence from UK higher education settings indicates that male students are significantly less likely to access university mental health services, even when experiencing substantial psychological distress [9].

Migration-related pressures may further intensify these gendered experiences. International students often encounter academic pressure, financial strain, language barriers, and social isolation, which have been associated with poorer mental health outcomes [4, 12]. For male students, expectations of academic success and future economic responsibility may amplify stress and contribute to elevated levels of depression and anxiety. Studies focusing on migrant and international student populations have similarly highlighted that male students may rely on avoidant coping strategies, increasing vulnerability to internalised distress [13].

The finding identifies male international students as a group warranting further investigation in comparable UK settings; it does not by itself justify a targeted intervention. Replication with larger and more gender-diverse samples, direct measurement of proposed mechanisms, and qualitative work on help-seeking would be needed before recommending gender-specific service redesign. In the interim, the result can inform cautious, non-stigmatising attention to whether existing services are equally accessible across gender groups.

### Regional differences in mental health outcomes

No statistically significant differences in depression, anxiety, or stress were detected across the regional groups. This should not be interpreted as evidence that the region is irrelevant or that groups are equivalent. The regional categories were broad and uneven, and within-region heterogeneity was not modelled. The null findings may therefore reflect limited statistical power, imprecise categorisation, or genuinely small differences. The most defensible conclusion is that this study did not detect regional variation under the present sampling and measurement conditions.

From an acculturation perspective, this finding is consistent with Berry’s acculturation model, which emphasises the role of acculturative stress and host-context factors in shaping mental health outcomes rather than cultural background alone [5, 6]. Research conducted in the UK and other Western higher education contexts has shown that factors such as academic pressure, language demands, financial strain, and social isolation tend to affect international students across regions in similar ways, potentially reducing observable regional variation in mental health outcomes [8, 14].

Methodological considerations may also help explain the absence of regional differences. Although participants represented multiple regions in the survey, group sizes were uneven, which may have limited statistical power to detect subtle differences between groups. Nevertheless, the findings align with studies suggesting that post-migration stressors and institutional environments exert a stronger influence on international students’ mental health than pre-migration cultural differences [6, 7].

### Length of stay and mental health outcomes

Length of stay was not significantly correlated with depression, anxiety, stress, or perceived mental stress. This result does not demonstrate that the duration of residence has no relationship with adjustment. Most participants had lived in the UK for 6–12 months, leaving limited variation and potentially obscuring non-linear or stage-specific patterns. In addition, coding ordered categories as a continuous variable assumes equal spacing between categories, an assumption that should be acknowledged. The finding is therefore best understood as a null association in this sample rather than evidence against temporal adaptation.

This finding is consistent with previous research, which indicates that adaptation to a new cultural and academic environment does not occur uniformly over time. Some [7, 8] studies have reported initial improvements in psychological well-being as students become familiar with their surroundings, followed by renewed stress related to academic demands, financial pressures, or uncertainty about prospects. As a result, the length of stay may have a limited or inconsistent relationship with mental health outcomes, particularly within relatively brief time frames such as those represented in the present sample.

From an acculturation perspective, Berry’s model emphasises that psychological outcomes may be more closely related to ongoing acculturative stressors than to duration of residence alone [5, 8]. Factors such as perceived discrimination, social connectedness, academic integration, and access to support services may play a more critical role in shaping mental health than the number of months spent in the host country. This may help explain why the length of stay did not emerge as a significant correlation of mental health outcomes in the present study.

### Predictors of depression

Perceived mental stress and gender were independently associated with depression scores in the regression model, whereas length of stay and region were not. The coefficient for perceived mental stress indicates a conditional statistical relationship, not an effect: students reporting greater stress also reported greater depressive symptoms after adjustment for the included covariates. Reverse directionality and common-method variance are plausible because stress and depression were measured at the same time using self-report instruments. Unmeasured factors, including financial hardship, social support, discrimination, prior mental-health history, and academic demands, may also account for part of the observed association.

Perceived mental stress emerged as the strongest statistical association with depression, highlighting the vital role of ongoing, subjective stressors influencing mental health outcomes. This finding is consistent with existing literature demonstrating that academic pressure, financial strain, uncertainty regarding future employment, and social isolation are key contributors to depression among international students [4, 14]. Evidence in the literature has also shown that perceived stress is often a stronger predictor of depressive symptoms than objective indicators of stress exposure, as it reflects how individuals appraise and cope with their circumstances [15, 16].

Gender also emerged as a significant predictor of depression after controlling for other variables. This finding aligns with studies suggesting that male international students may be particularly vulnerable to internalised distress due to gendered norms surrounding emotional expression and help-seeking behaviours [11]. Within migrant and international student populations, cultural expectations related to masculinity, academic success, and economic responsibility have been associated with increased psychological strain and reduced engagement with mental health services [5, 15].

In contrast, length of stay and region of origin did not significantly predict depression in the regression model. This is consistent with evidence suggesting that mental health outcomes among international students may be more closely related to ongoing acculturative stressors and contextual factors than to duration of residence or cultural background alone [5, 6, 8]. From an acculturation perspective, these findings reinforce the view that adjustment is not a simple linear process over time, but rather one shaped by the interaction between individuals and their host environment.

The regression findings justify prioritising perceived stress and gender as hypotheses for further study, not as confirmed intervention targets. Future analyses should test robustness using validated acculturative-stress measures, theoretically specified confounders, larger samples, and longitudinal designs capable of establishing temporal ordering.

### Strengths and limitations

A strength of the study is its focus on international students in a post-1992 UK university, a setting less frequently represented in published quantitative research. The use of the DASS-21 permits comparison with other studies, and the analysis considers several migration-related and demographic variables simultaneously. These strengths support the study’s value as context-specific, hypothesis-generating evidence, but they do not overcome the design and measurement limitations described below.

The analytical strategy combined descriptive, group-comparison, correlational, and regression methods. However, the number of analyses increases the possibility of chance findings, and no adjustment for multiple testing was reported. Effect sizes and confidence intervals were not consistently presented, limiting assessment of precision and practical importance. The regression model also included categorical variables whose coding and reference categories should be stated explicitly, particularly for gender and region.

The cross-sectional design precludes causal inference and temporal ordering. Associations between perceived stress and depression may be bidirectional, and both may reflect unmeasured common causes. Longitudinal or experimental designs would be required to examine whether changes in stress precede changes in mental health. Therefore, terms such as impact, influence, effect, and predictor are avoided where they could imply causation; regression variables are described as statistically associated with the outcome.

Convenience sampling creates a substantial risk of selection bias because students experiencing either greater distress or greater engagement with university networks may have been more likely to participate. The sample size was modest, no a priori power calculation was reported, and subgroup sizes were uneven. The broad regional categories may mask important within-region differences, while the concentration in Public Health programmes and the limited variation in reason for migration and length of stay constrain external validity. Self-report data are also vulnerable to recall, social desirability, and common-method bias.

The study was conducted at a single institution and cannot be assumed to represent international students across UK higher education. The study-specific perceived mental-stress measure requires fuller psychometric evaluation, including factor structure and validity; internal consistency alone is insufficient. In addition, potentially important confounders—such as age, level of study, socioeconomic position, prior mental-health history, English-language proficiency, discrimination, social support, and current academic or visa pressures- were not included in the model. These limitations mean that the findings should be used to formulate and refine research questions rather than to estimate population prevalence or prescribe policy.

### Implications for research

The principal implication of this study is research. The observed association between perceived mental stress and depression should be tested in adequately powered, multi-institutional longitudinal studies using validated measures and prespecified causal models. Such studies should distinguish specific stress domains, assess baseline mental health, and measure plausible confounders and mediators. The unexpected gender pattern also requires replication and direct investigation of mechanisms rather than attribution to gender itself.

The null findings for length of stay and region should similarly guide study design rather than be treated as evidence of no relationship. Future research should recruit participants across wider residence durations, model time non-linearly, use more meaningful cultural and migration indicators, and report effect sizes with confidence intervals. Qualitative or mixed-methods work could examine how students interpret stressors and support, thereby improving the content validity of future measures.

Any practice implications must remain provisional. Within the University of Wolverhampton, the findings may justify reviewing whether existing, broadly available support is accessible to international students and whether stress-related concerns are routinely assessed. However, this study cannot establish that a particular service or policy will improve outcomes, and it does not provide comparative evidence for a gender-targeted or stress-focused intervention. Decisions about service changes should therefore draw on stronger evidence, local needs assessment, student consultation, and prospective evaluation.

## Conclusion

This exploratory cross-sectional study found that perceived mental stress and gender were statistically associated with depression scores among 115 international students at one UK university, while region of origin and length of stay were not. Men also reported higher mean depression, anxiety, and stress scores than women. These results describe relationships within the sample; they do not establish causal effects, population prevalence, or equivalence between groups with non-significant differences.

Interpreted through Berry’s acculturation framework, the findings are consistent with the proposition that the appraisal of current post-migration demands may be more closely related to mental health than distal indicators such as residence duration or broad region of origin. However, the study did not directly test the full framework or measure several key contextual and coping processes. The framework, therefore, supports interpretation and hypothesis development rather than confirmation of a causal pathway.

The study contributes context-specific, hypothesis-generating evidence and identifies priorities for further research: replication in larger and more diverse samples; longitudinal assessment of temporal ordering; improved measurement of acculturative stress; explicit testing of gender-related mechanisms; and fuller control of confounding. Until such evidence is available, policy and practice conclusions should remain cautious. Universities may use the findings to inform local enquiry and evaluation, but not as proof that a specific intervention will improve mental health outcomes.

## Supplementary Information

Supplementary Material 1.

## Data Availability

The original contributions presented in the study are included in the article/ supplementary material, futher inquiries can be directed to the corresponding author.
