# Supplementary material for: Migration-related factors and mental health among international students in a UK higher education setting: a cross-sectional study
Source: BMC Public Health. 2026 Aug 1;26:3326. doi: 10.1186/s12889-026-28749-4 (PMC13753085; doi:10.1186/s12889-026-28749-4)
Supplement: Supplementary file 1 [file 12889_2026_28749_MOESM1_ESM.docx]

Anonymised dataset

anonymised dataset used for analysis.

SPSS Output (Selected Screenshots)

Figure B1: Descriptive statistics

Independent Samples t-test

One-way ANOVA

Correlation

Regression coefficients

Survey Instrument (Selected Screenshots)

Screenshot of DASS-21 items used in the online questionnaire and response scale

DASS-21 items (16-17)

migration-related stress questions
